# Australian postpartum women want reputable health information delivered via social networking sites

**DOI:** 10.1111/ajo.13840

**Published:** 2024-05-22

**Authors:** Megan L. Gow, Maddison Henderson, Amanda Henry, Lynne Roberts, Heike Roth

**Affiliations:** ^1^ Discipline of Paediatrics and Child Health School of Clinical Medicine, UNSW Medicine and Health Sydney New South Wales Australia; ^2^ The University of Sydney Children's Hospital Westmead Clinical School Sydney New South Wales Australia; ^3^ Department of Women's and Children's Health St George Hospital Sydney New South Wales Australia; ^4^ Institute of Endocrinology and Diabetes, The Children's Hospital at Westmead Sydney New South Wales Australia; ^5^ Discipline of Women's Health, School of Clinical Medicine UNSW Medicine and Health Sydney New South Wales Australia; ^6^ St George and Sutherland Clinical Campus, School of Clinical Medicine UNSW Medicine and Health Sydney New South Wales Australia; ^7^ Faculty of Health University of Technology Sydney Sydney New South Wales Australia

**Keywords:** health promotion, maternal health, postpartum period, social media, social networking

## Abstract

Novel strategies are needed to target the health of postpartum women, who face numerous competing demands. This survey study of 553 postpartum women found 90% want access to a range of health information via social networking sites from reputable professionals, with Instagram (71%) the preferred platform. Delivery of health information to postpartum women via health organisation social networking sites could deliver cost savings and health benefits for postpartum women.

## INTRODUCTION

Health of women during the postpartum period is key for influencing the future health of mothers, infants, and subsequent pregnancies.^1^ However, postpartum women face a barrage of competing demands,^2^ meaning their own health may not take priority. Therefore, novel strategies are needed to target the health of this population.

Social networking platforms are highly accessed by women of reproductive age. For example, 39% of the billion monthly active Instagram users are females aged 18–44 years.^3^ Given their convenience, accessibility, and low cost, social networking sites provide a plausible avenue for delivering health information to postpartum women. However, it is unknown whether postpartum women want to receive health information on social networking sites. This study investigated whether social networking sites are an acceptable platform for conveying health information to postpartum women.

## MATERIALS AND METHODS

Women eligible to participate in this survey study were Australian‐residing, English speaking, and aged ≥18 years, and had given birth within the last two years, had one or more social networking accounts, and internet access. This study was approved by The University of Sydney's Human Research Ethics Committee (protocol number: 2022/148).

Utilising snowball sampling, recruitment occurred from August to November 2022 via social networking site advertisements, Playgroup NSW newsletters and paid text message advertisements to Playgroup NSW members. Survey responses were non‐identifiable.

All participants completed a questionnaire online using REDCap, including key demographics (eg age, ethnicity, education, relationship status, number of children), health status (eg height, weight, current health risks, pregnancy complications), and social networking site use (accounts, usage, contacts).

Participants were asked: whether and what health information they had previously accessed on social networking sites; whether and what health information they would like access to on social networking sites and which sites; and how they would like health information communicated to them.

Descriptive analysis was conducted using SPSS v28.0 (SPSS Statistics for Windows, Armonk, NY, USA).

## RESULTS

Table 1 shows participant characteristics of the 553 women with completed questionnaires. Most were aged 26–35 years, had a university and/or postgraduate degree, and approximately three‐quarters identified as Caucasian. Almost all were in a relationship, and approximately half had one child.

**Table 1:** Characteristics of 553 study participants

**Age group, *n* (%)**	
18–25	15 (3)
26–35	370 (67)
36–45	166 (30)
46–55	2 (0)
**Ethnicity, *n* (%)**	
Asian	63 (11)
Caucasian	410 (74)
European	49 (9)
Aboriginal and/or Torres Strait Islander	9 (2)
Polynesian or Māori	5 (1)
Mixed race	4 (1)
Prefer not to say	5 (1)
Other	8 (1)
**Education, *n* (%)**	
Secondary school	50 (9)
Trade certificate/ diploma	107 (19)
University degree	215 (39)
Postgraduate degree	178 (32)
Prefer not to say	2 (0)
Other	1 (0)
**In a relationship, n (%)**	536 (97)
**Recruitment from, n (%)**	
Friend	27 (5)
Text message from PNSW	56 (10)
PNSW Newsletter	174 (32)
PNSW social media post	14 (3)
Other social media	277 (50)
Other	5 (1)
**Number of children (5 missing), n (%)**	
1	270 (49)
2	203 (37)
3	60 (11)
4	15 (3)
**Age of youngest child, n (%)**	
<6 months	124 (22)
6–12 months	175 (32)
13–24 months	254 (46)
**Pregnant**	25 (5)
**BMI, mean ± SD (12 missing)**	26.8 ± 6.6
**Health risk present, n (%)**	
Smoking	13 (2)
Obesity	119 (22)
High alcohol consumption	15 (3)
High cholesterol	14 (3)
High blood pressure	16 (3)
Diabetes	5 (1)
None	385 (60)
Other	10 (2)
**Pregnancy conditions, *n* (%)**	
HDP	56 (10)
GDM	105 (19)
T1D or T2D	4 (1)
EGWG	78 (14)
None	319 (58)
Other^†^	25 (5)

^†^ Most ‘Other’ pregnancy complications were the condition hyperemesis gravida.

BMI, body mass index; EGWG, excessive gestational weight gain; GDM, gestational diabetes mellitus; HDP, hypertensive disorder of pregnancy; *n*, number; PNSW, Playgroup NSW; SD, standard deviation; T1D, type 1 diabetes; T2D, type 2 diabetes.

### Social networking use and preferences

Overall, 95% had a Facebook, 92% Instagram, 16% Twitter, 36% LinkedIn and 22% TikTok account (Table 2). Participants accessed Facebook and Instagram more regularly (82% and 81%, respectively reporting use more than once a day) and had more connections/followers/friends on these platforms.

**Table 2:** Participant social networking account use and connections

	Facebook *n* = 523	Instagram *n* = 508	Twitter *n* = 91	LinkedIn *n* = 200	TikTok *n* = 123
**Account usage**					
**More than once per day**	431 (82)	412 (81)	17 (19)	11 (6)	51 (41)
2–5 times per day	173 (33)	146 (29)	8 (9)	9 (5)	24 (20)
6–10 times per day	145 (28)	143 (28)	5 (5)	1 (1)	13 (11)
>10 times per day	113 (22)	123 (24)	4 (4)	1 (1)	14 (11)
**Once per day**	44 (8)	46 (9)	7 (8)	18 (9)	13 (11)
**A few times per week but not daily**	32 (6)	35 (7)	17 (19)	50 (25)	24 (20)
**Once per week**	4 (1)	7 (1)	8 (9)	27 (14)	12 (10)
**Less than once per week**	12 (2)	8 (2)	42 (46)	93 (47)	23 (19)
**Account followers/ friends/ connections**					
0–10	4 (1)	22 (4)	36 (40)	10 (5)	78 (63)
10–50	13 (2)	46 (9)	10 (11)	34 (17)	23 (19)
50–100	58 (11)	101 (20)	8 (9)	41 (21)	6 (5)
100–500	317 (61)	275 (54)	19 (21)	77 (39)	13 (11)
500–1000	90 (17)	43 (8)	6	21 (11)	2 (2)
1000–2000	31 (6)	7 (1)	5	5 (3)	0 (0)
More than 2000	3 (1)	10 (2)	3	3 (2)	0 (0)
Prefer not to say	7 (1)	4 (1)	4	9 (5)	1 (1)

All data expressed as number (percentage). *n*, number.

Overall, 87% of women reported previously accessing health information on social networking sites and 90% wanted future access. Instagram (71%) and Facebook (53%) were the preferred platforms. Only 5%, 3% and 1% of participants wanted access to health information on TikTok, Twitter and LinkedIn, respectively.

Participants identified mental health (87%), breastfeeding (73%), infant feeding (65%), maternal nutrition (60%) and maternal physical activity (52%) as important health issues for women with infants under two years of age. In total, 513 participants indicated how likely they would be to access a variety of health information on social networking sites. Approximately three‐quarters of participants were highly/somewhat likely to access all types of health information (Table 3), including breastfeeding, maternal nutrition, maternal physical activity, mental health, infant feeding and information about specific health conditions. Participants also indicated their preference for information delivery via health professionals (87%), health organisations (83%) and researchers (54%), rather than via influencers (9%) or marketing of products (7%).

**Table 3:** Likelihood of accessing various types of information on social networking platforms

	Breastfeeding *n* = 513	Maternal nutrition *n* = 513	Maternal physical activity *n* = 513	Mental health *n* = 513	Infant feeding *n* = 513	Specific health condition *n* = 513
Highly likely	205 (40)	174 (34)	167 (33)	189 (37)	246 (48)	151 (29)
Somewhat likely	168 (33)	210 (41)	205 (40)	200 (39)	183 (36)	183 (36)
Somewhat unlikely	56 (11)	58 (11)	56 (11)	46 (9)	29 (6)	57 (11)
Highly unlikely	62 (12)	32 (6)	40 (8)	26 (5)	32 (6)	47 (9)
Not sure	22 (4)	39 (8)	45 (9)	52 (10)	23 (4)	75 (15)

All data expressed as number (percentage). *n*, number.

## DISCUSSION

Our findings demonstrate that postpartum women want access to a variety of health information on social networking platforms, specifically Facebook and Instagram. In fact, similar to a preconception study,^4^ the majority (80%) of women in our study were already utilising social networking sites to access health information. Although research in this area is limited, the evidence to date suggests that this preference for access to health information on social networking sites is largely unmet, with a recent content analysis of the hashtag #postpartumbody finding that only 9% of images uploaded to Instagram linked to this hashtag were text‐focused, including health messaging.^5^

Women surveyed in our study indicated they wanted health information to be provided by reputable sources, highlighting the need for healthcare professionals and researchers to disseminate health information via this avenue. However, there are documented challenges in disseminating health information via social networking sites. Interviews with Australian researchers and health promotion experts revealed barriers to providing health information via technology include competing priorities, resource limitations, organisational capacity and the challenge that technology develops more rapidly than the time taken to develop and ready evidence for translation.^6^ Our research suggests that healthcare providers need to overcome these barriers to provide a service that is wanted by postpartum women and may be key to delivering timely health care to this population. Women also wanted access to a range of health information on social networking sites, with mental health identified as the most important health issue. This supports the need to provide information that is varied and holistic.

A 2023 scoping review^7^ of health promotion interventions delivered to women of reproductive age via social networking sites identified three studies^8^, ^9^, ^10^ assessing the effectiveness of interventions specifically to postpartum women. These studies, none conducted in Australia, highlighted the effectiveness of social media‐delivered health promotion to improve a range of knowledge and health outcomes for postpartum women and infants with higher engagement compared with face‐to‐face interventions. None of these previous studies utilised Instagram, which was the preferred platform for health information delivery nominated by our participants. Future interventions need to consider that social networking site popularity and usage patterns are constantly evolving. Intervention design will need to monitor these patterns and adapt interventions to suit the most accessible sites.

While social networking sites provide an avenue for disseminating widespread accessible health information, dissemination of general postpartum health information by this mode may not be suitable for certain subgroups. This is supported by research demonstrating that cultural and socio‐economic status differences in individuals influence the reasons for engaging with the social networking site, Instagram, including their likelihood to access information via this avenue.^11^ Various subgroups of women, including non‐English speaking women, women of different cultures, women who experienced pregnancy complications, and women experiencing social issues such as poverty or abuse, are likely to have differing healthcare needs and priorities unique to their situation. Indeed, while there are several strengths to our research, including recruitment of a large sample and narrow inclusion criteria, limitations include a highly educated, mostly Caucasian sample of women who had to hold one or more social networking site accounts to be eligible, reducing generalisability of our findings. Further research is required to understand how to reach all marginalised groups with postpartum health information that is relevant to their specific needs.

In conclusion, it is apparent that postpartum women want access to health information on social networking sites, delivered by qualified health professionals or researchers. Further research, including collaboration with the health promotion sector, should explore implementation of cost‐effective interventions delivered via social networking sites, designed to educate postpartum women and holistically optimise their physical and psychological health.
